# Preoperative predictors for a successful return to sport following anterior cruciate ligament reconstruction (ACLR): a protocol for a systematic review and meta-analysis

**DOI:** 10.1136/bmjopen-2020-048295

**Published:** 2021-12-23

**Authors:** Hayley Carter, Gwyn Lewis, Benjamin Edward Smith

**Affiliations:** 1 Department of Physiotherapy, University Hospitals of Derby and Burton NHS Foundation Trust, Derby, UK; 2 Department of Physiotherapy, Health and Rehabilitation Research Institute, Auckland University of Technology, Auckland, New Zealand; 3 Division of Rehabilitation, Ageing and Wellbeing, University of Nottingham, Nottingham, Nottinghamshire, UK

**Keywords:** orthopaedic sports trauma, musculoskeletal disorders, knee, orthopaedic & trauma surgery

## Abstract

**Introduction:**

Return to sport (RTS) following anterior cruciate ligament reconstruction (ACLR) is the primary goal for most patients. However, the decision of when to RTS is difficult for patients and clinicians as it is based on limited available evidence. Over the past decade, a number of predictor variables have been associated with RTS after ACLR. We present a Preferred Reporting Items for Systematic Reviews and Meta-Analysis Protocols compliant protocol for a systematic review and meta-analysis of preoperative predictors for RTS/preinjury levels of physical activity following ACLR.

**Methods and analysis:**

A literature search will be performed in six electronic databases (CINAHL, AMED, MEDLINE, SPORTDiscus and PsycINFO via EBSCOhost and Web of Science) from inception to December 2020. Prospective, retrospective and cross-sectional study designs will be included. To be included, studies will need to identify at least one preoperative predictor and identify the relationship between the predictor(s) and RTS/preinjury levels of physical activity following ACLR. Blinded assessment with consensus agreement will be applied for inclusion of studies, data extraction, risk of bias assessment using the Quality in Prognostic Studies tool and the Grading of Recommendations Assessment, Development and Evaluation framework. If data allows and studies are considered homogeneous, data will be pooled through a meta-analysis. If heterogenous, a narrative synthesis will be completed. Subgroup and sensitivity analyses will be completed, where appropriate.

**Ethics and dissemination:**

Ethical permission is not required for this study. The results will be published in a peer-reviewed journal and presented at national and international conferences.

**PROSPERO registration number:**

CRD42020222567.

**Strengths and limitations of this study**

- A strength of this review is that it aims to complete a meta-analysis of prospective, retrospective and cross-sectional study designs, thus adopting an inclusive approach.
- An extensive search will be completed in a number of electronic databases in addition to searching the reference lists of included studies to maximise search outputs.
- By completing a thorough risk of bias assessment, results will be discussed with transparency, and the certainty of the evidence will be assessed through the use of the standardised Grading of Recommendations Assessment, Development and Evaluation framework.
- Omitting studies not published in the English language may contribute to limitations related to publication bias.
- The broad approach to the outcome of interest (return to sport/preinjury levels of physical activity) with no restriction of a time point of interest may present challenges for data syntheses.

## Introduction

Return to sport (RTS)/physical activity following anterior cruciate ligament reconstruction (ACLR) remains a complex clinical problem. Following a lengthy rehabilitation process for both patients and clinicians, the minimum time for RTS is generally considered 9 months following surgery.^1^ However, it is recommended that the decision is based on progression through objective criteria rather than time alone,^2 3^ with a 2016 consensus statement suggesting that RTS should be assessed on a continuum rather than a set point, with the decision shared between all stakeholders.^4^

There is currently a lack of validated guidelines for ACL rehabilitation and RTS criteria, and, therefore, best practice is unknown.^5–8^ Additionally, there is not always a clear relationship between outcomes of RTS testing and actual RTS. For example, many patients return to their preinjury activity in spite of failing RTS criteria,^9^ while others pass RTS criteria but do not make a full RTS. A number of tests are often included in RTS criteria that are combined to form a ‘test battery’, with studies reporting between 10 and 20 different tests.^5 10^ It is, therefore, unsurprising that the proportion of patients who pass all these test battery components is low.^5^ Other studies have indicated that psychological factors, rather than physical, may limit RTS following ACLR.^11^ Clinically, these points raise concerns about how RTS testing can best be used.

Returning to sport is the primary goal for most patients following surgery,^4 12 13^ who often assume this to be a realistic goal.^14^ However, recent studies documenting RTS/preinjury activity level outcomes report suboptimal outcomes following surgery with only 24% returning at 1 year,^15^ less than 45% returning at 2 years^16 17^ and 62% returning at 5 years.^18^ It is, therefore, clear that we have further work to do to optimise the care and outcomes for this patient population to meet their expectations of surgery and rehabilitation.

One way to improve RTS outcomes may be to identify preoperative predictive variables associated with RTS or preinjury levels of physical activity. This would allow clinicians to better address patient expectations prior to surgery and facilitate future research to develop interventions targeted at these predictive variables, improving RTS outcomes. Over the last decade, a number of risk factors have been identified with the failure to RTS following ACLR, including functional markers (eg, muscle strength and single leg hop scores), patient-reported outcomes, psychological responses and person-related factors (eg, age, gender and motivation levels).^17 19–23^ However, no study to date has collated the available evidence to identify preoperative predictors for failure to RTS. Thus, a new synthesis of the literature is warranted to help inform patients, clinicians and researchers about risk factors for poor RTS outcomes following ACLR.

### Objectives

The aim of this review is to synthesise the available data to determine the preoperative predictors for a successful RTS/preinjury levels of physical activity after ACLR.

## Methods and analysis

This systematic review protocol was drafted using the International Prospective Register of Systematic Reviews (PROSPERO) as a guideline and registered in PROSPERO (2 December 2020, https://www.crd.york.ac.uk/prospero/display_record.php?). Any changes made to the protocol will be updated in PROSPERO. The protocol was prepared in accordance with the Preferred Reporting Items for Systematic reviews and Meta-Analysis Protocols (PRISMA-P) checklist (online supplemental file 1).^24 25^

10.1136/bmjopen-2020-048295.supp1 Supplementary data

### Eligibility criteria

The eligibility criteria are prespecified by the Population-Exposure-Outcome-Study design and are described below.

#### Population

The population of focus will be adults aged 18–65, who have undergone a primary ACLR. However, studies will be included where participants are <18, but the mean age of the overall population is ≥18, as the authors acknowledge that a large proportion of ACL ruptures occur in adolescents. However, the main population of interest is adults, as this review is preparatory work that will contribute to intervention development for adults in a National Health Service (NHS) clinical pathway.

#### Exposure

To be included, studies will need to identify at least one potential preoperative predictor variable and identify the relationship between the predictor(s) and RTS/preinjury levels of physical activity. All estimates considered to determine the relationship between the predictive factor and outcome of interest will be included (eg, OR and p value). Predictive factors may be demographic (eg, age), physical (eg, quadriceps strength) or psychosocial (eg, anxiety). We aim to include any identified predictor variable in the review as available. That is, both those that do and do not have a significant relationship with the outcome of interest.

#### Outcome

The main outcome of interest is the success of RTS or preinjury levels of physical activity. The identified preoperative risk factors should be linked to the outcome of interest. No time limit has been defined for the reported outcome. All measures of RTS/preinjury levels of physical activity will be included (eg, participant reported [yes/no] or validated measures [Tegner, Marx scale], this list is, however, not exhaustive).

#### Study

This review will include human studies in the English language with full texts available. Prospective, retrospective and cross-sectional study designs will be included.

### Review question

The review question is ‘what preoperative factors predict a RTS or preinjury level of physical activity following ACLR?’

### Timeline

The timeline for this study is presented in table 1. The research question has been specified, protocol details have been registered and published via PROSPERO, the search has been started and formal screening of the search results is in progress.

**Table 1:** Study timeline

Review stage	Element	Status
Review question	PEOS determined	Completed November 2020
Register review	PROSPERO	Completed December 2020
Search strategy	Literature search in electronic databases and reference lists	Ongoing
Study selection	Title, abstract and full-text review	Ongoing
Data extraction	Data extraction form	Ongoing
Risk of bias	See figure 1	Ongoing
Certainty of evidence	GRADE	Planned
Analysis	Narrative/meta-analysis (sensitivity and sub-group analysis as appropriate)	Planned
Publication	Journals and conferences (international and national)	Planned

GRADE, Grading of Recommendations Assessment, Development and Evaluation; PEOS, Population-Exposure-Outcome-Study; PROSPERO, Prospective Register of Systematic Reviews.

**Figure 1:**
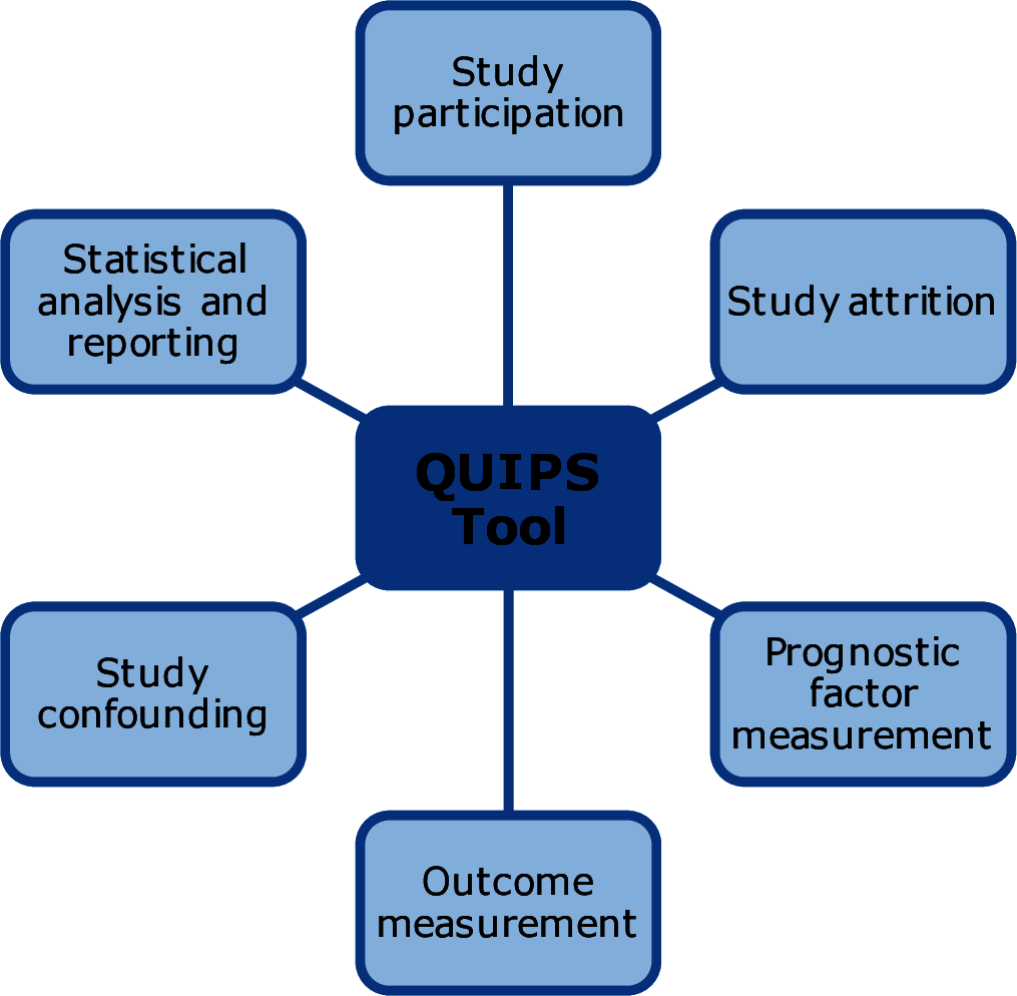
Six domains assessed in the QUIPS tool. QUIPS, Quality in Prognostic Studies.

### Search strategy

A systematic search will be conducted in six electronic databases (CINAHL, AMED, MEDLINE, SPORTDiscus and PsycINFO via EBSCOhost and Web of Science) from inception to December 2020. Reference lists of included articles will also be examined. The search strategy includes a combination of key words in four categories: (1) ACL, (2) preoperative, (3) risk factors and (4) RTS/preinjury levels of physical activity. Terms will be matched to Medical Subject Headings (MeSH) where appropriate and combined used Boolean operators. An example of the search strategy is included in online supplemental file 2.

10.1136/bmjopen-2020-048295.supp2 Supplementary data

### Data collection and analysis

#### Data management and selection

Following the search in the listed databases, references will be exported to a referencing management software where duplicates will be removed.

Titles will be screened against the eligibility criteria by one reviewer (HC). The same reviewer will then screen abstracts for full-text review. Two reviewers (HC and BES) will independently screen full-text articles for inclusion against the eligibility criteria. Agreement will be discussed to reach consensus with a third reviewer (GL) available to solve any discrepancies where consensus cannot be reached. Reference lists of the included articles will be screened independently and agreed for inclusion following the same process as above. A PRISMA flow diagram will be used to document the selection process along with reasons for exclusion.^26^

#### Data extraction

Data extraction will be completed independently by one reviewer (HC) and verified by a second reviewer against the following domains: author, year of publication, study design, sample size, participant details, preoperative predictors and their relationship with RTS or preinjury level of physical activity (eg, OR and p value), outcome measure used and time point of reported outcome. If additional data are needed about a particular study, the corresponding author will be contacted to obtain the required detail.

### Quality assessment

A quality assessment will be carried out on all studies independently by two reviewers (HC and BES). The Quality in Prognostic Studies (QUIPS) tool is suggested to assess risk of bias in prognostic factor studies.^27–29^ It comprises of six domains with a number of facilitatory questions in each to allow a rating of low, moderate and high risk of bias to be made. The six domains assessed are shown in figure 1 with the full QUIPS tool available in online supplemental file 3.

10.1136/bmjopen-2020-048295.supp3 Supplementary data

Disagreements between the review authors regarding the risk of bias in particular studies will be resolved by discussion, with the involvement of a third review author (GL) if necessary.

When the risk of bias assessment is complete, the level of consensus will be evaluated using Cohen’s kappa statistic as follows: (1) none to slight 0.01–0.2, (2) fair 0.21–0.4, (3) moderate 0.41–0.6, (4) substantial 0.61–0.8, (5) almost perfect 0.81–1.00.^30^ All studies that met the inclusion criteria will be included in the review regardless of methodological quality. The methodological quality of the included studies will be considered in the interpretation and discussion of the results.

### Sensitivity analysis

Where relevant, a sensitivity analysis will be completed to ensure that results are interpreted and discussed appropriately. Studies judged to be at high risk of bias will be excluded and compared with the meta-analysis results where all studies are included. Further sensitivity analysis may be completed as appropriate, where the meta-analysis will be rerun according to study design (eg, prospective and retrospective methodology) and time point for return (eg, 1 year and 2 years).

### GRADE

The certainty of evidence for each prognostic factor will be derived using the Grading of Recommendations Assessment, Development and Evaluation (GRADE) framework.^31 32^ The overall certainty of evidence will be rated as high, moderate, low or very low. GRADE ratings will be assigned by two reviewers (HC and BES) and disagreements will be resolved through consensus with a third reviewer available as needed (GL).

### Data synthesis strategy

Clinical heterogeneity will be assessed through visual examination of the data extraction table on details related to participant characteristics (eg, sex, age), risk factors (eg, Anterior Cruciate Ligament Return to Sport After Injury [ACL-RSI] score, laxity, physical activity level and smoking [list not exhaustive]), RTS/preinjury levels of physical activity outcomes (eg, participant reported [yes/no] or validated measures [Tegner, Marx scale]), data points (eg, data collected at 6 months postsurgery, 12 months or 2 years), study design (eg, retrospective or prospective) and process (eg, retrospective data analysed from an existing database or prospective study carried out at orthopaedic clinic) in the included studies. The data extraction table will be independently verified by a second reviewer. If heterogeneous, data will be assessed narratively.

If at least two studies are deemed to be capturing the same risk factor within similar populations, statistical heterogeneity will be assessed using the I^2^ statistic where 0%–25% is low, 26%–74% is moderate and 75% and over high statistical heterogeneity.^33^ If data allow and studies are considered homogenous, data will be pooled through a meta-analysis. The random effects model will be used for high statistical heterogeneity^34^ and a fixed effects model for low statistical heterogeneity.^35^

Publication bias will be assessed by an asymmetry test.^36^ All data analysis will be performed using the OpenMetaAnalyst software.^37^

### Subgroup analysis

Subgroup analysis may be performed, if possible, according to the predictors associated with a RTS compared with those associated with a return to preinjury level of physical activity. It is acknowledged that there is a lack of consensus regarding the terminology used when reporting return to physical activity outcomes.^5^ Based on our scoping exercise, studies frequently report this outcome differently (eg, RTS, return to preinjury levels of physical activity and return to performance), and this has, therefore, been considered by the research team to ensure that all relevant literatures are reviewed. Further analysis may be performed, as appropriate, to compare population groups (eg, athletes vs recreational participants).

### Patient and public involvement

No patient or public involvement is planned for the design and execution of this review. However, patient/public participation may be sought to aid with dissemination of the review findings.

As this review will only be focusing on the currently published literature, ethics approval is not required. Results from this systematic review will be published in a peer-reviewed journal. Where relevant, the results will also be presented at appropriate national and international conferences.

## Discussion

The present review will use rigorous methodology to provide a review of preoperative predictors for successful RTS/physical activity following ACLR. The findings will help improve research and clinical practice, enabling highlighted risk factors to be further explored and understood. Acknowledging pertinent prognostic indicators prior to surgery will allow clinicians to begin exploring and investigating these with patients to improve education regarding the surgical pathway and provide a clearer indication for returning to sport or preinjury levels of physical activity. The results will also help direct future research towards the development of targeted interventions for identified predictors with aim to improve RTS success.

As a result of the risk of bias assessment and GRADE approach, our review may also identify the strengths and limitations of research in this field and provide insights into future research areas. Findings will be disseminated widely to maximise the impact of the results to help a wide audience of patients, healthcare professionals and all other relevant stakeholders.

### Strengths and limitations

A strength of this review is that it aims to complete a meta-analysis of prospective, retrospective and cross-sectional study designs, thus adopting an inclusive approach. An extensive search will be completed in a number of electronic databases in addition to the reference lists of included studies to maximise search outputs. By completing a thorough risk of bias assessment that has been customed to the aims of the review, results will be discussed with transparency. Further certainty of the evidence will be assessed using the GRADE approach.

The review will omit studies not published in the English language, which may contribute to limitations related to publication bias.

## Data Availability

All data relevant to the study are included in the article or uploaded as supplementary information. Study results of the systematic review will be published in another article.
