# Supplementary material for: Preoperative predictors for a successful return to sport following anterior cruciate ligament reconstruction (ACLR): a protocol for a systematic review and meta-analysis
Source: BMJ Open. 2021 Dec 23;11(12):e048295. doi: 10.1136/bmjopen-2020-048295 (PMC8712982; doi:10.1136/bmjopen-2020-048295)
Supplement: Supplementary data [file bmjopen-2020-048295supp002.pdf]

Supplementary File 2 – Search Strategy

**Category**	ACL	Preoperative	Prognostic factors	RTS/preinjury level
**MeSH Heading**	Anterior cruciate ligament	Preoperative Period	Risk factors	Return to sport
**Alternatives**		Preop*	Risk	Return to preinjury level
		Pre-op*	Predictor	Return to physical activity
		Periop*	Prognostic	Return to Performance

All terms in each category will be combined with OR. All terms across categories will be combined with AND as follows:

1. Anterior cruciate ligament
2. ACL
3. 1 OR 2
4. Preoperative period
5. Preop*
6. Pre-op*
7. Periop*
8. 5 OR 6 OR 7
9. Risk factors
10. Risk
11. Predictor
12. Prognostic
13. 9 OR 10 OR 11 OR 12
14. Return to sport
15. Return to preinjury level
16. Return to physical activity
17. Return to performance
18. 14 OR 15 OR 16 OR 17
19. 3 AND 8 AND 13 AND 18
