# Supplementary material for: Preoperative predictors for a successful return to sport following anterior cruciate ligament reconstruction (ACLR): a protocol for a systematic review and meta-analysis
Source: BMJ Open. 2021 Dec 23;11(12):e048295. doi: 10.1136/bmjopen-2020-048295 (PMC8712982; doi:10.1136/bmjopen-2020-048295)
Supplement: Supplementary data [file bmjopen-2020-048295supp003.pdf]

Supplementary File 3 – QUIPS Tool

QUIPS Risk of Bias Assessment Instrument for Prognostic Factor Studies

Modified from: Hayden JA, Côté P, Bombardier C. Evaluation of the Quality of Prognosis Studies in Systematic Reviews. Annals of Internal Medicine. 2006;144:427-437, with the assistance of the QUIPS-LBP Working Group.

**Author and year of publication**				
**Study identifier**				
**Reviewer**				
**Biases**	**Issues to consider for judging overall rating of "Risk of bias"**	**Study Methods & Comments**	**Rating of reporting**	**Rating of "Risk of bias"**
Instructions to assess the risk of each potential bias:	These issues will guide your thinking and judgment about the overall risk of bias within each of the 6 domains. Some 'issues' may not be relevant to the specific study or the review research question. These issues are taken together to inform the overall judgment of potential bias for each of the 6 domains.	Provide comments or text exerpts in the white boxes below, as necessary, to facilitate the consensus process that will follow.	Click on each of the blue cells and choose from the drop down menu to rate the adequacy of reporting as yes, partial, no or unsure.	Click on the green cells; choose from the drop-down menu to rate potential risk of bias for each of the 6 domains as High, Moderate, or Low considering all relevant issues
**1. Study Participation**	**Goal: To judge the risk of selection bias (likelihood that relationship between *PF* and *outcome* is different for participants and eligible non-participants).**			
Source of target population	The source population or population of interest is adequately described for key characteristics (LIST).			
Method used to identify population	The sampling frame and recruitment are adequately described, including methods to identify the sample sufficient to limit potential bias (number and type used, e.g., referral patterns in health care)			
Recruitment period	Period of recruitment is adequately described			
Place of recruitment	Place of recruitment (setting and geographic location) are adequately described			
Inclusion and exclusion criteria	Inclusion and exclusion criteria are adequately described (e.g., including explicit diagnostic criteria or "zero time" description).			
Adequate study participation	There is adequate participation in the study by eligible individuals			
Baseline characteristics	The baseline study sample (i.e., individuals entering the study) is adequately described for key characteristics (LIST).			

**Summary Study participation**	**The study sample represents the population of interest on key characteristics, sufficient to limit potential bias of the observed relationship between PF and outcome.**			
**2. Study Attrition**	**Goal: To judge the risk of attrition bias (likelihood that relationship between *PF* and *outcome* are different for completing and non-completing participants).**			
Proportion of baseline sample available for analysis	Response rate (i.e., proportion of study sample completing the study and providing outcome data) is adequate.			
Attempts to collect information on participants who dropped out	Attempts to collect information on participants who dropped out of the study are described.			
Reasons and potential impact of subjects lost to follow-up	Reasons for loss to follow-up are provided.			
Outcome and prognostic factor information on those lost to follow-up	Participants lost to follow-up are adequately described for key characteristics (LIST).			
	There are no important differences between key characteristics (LIST) and outcomes in participants who completed the study and those who did not.			
**Study Attrition Summary**	**Loss to follow-up (from baseline sample to study population analyzed) is not associated with key characteristics (i.e., the study data adequately represent the sample) sufficient to limit potential bias to the observed relationship between PF and outcome.**			
**3. Prognostic Factor Measurement**	**Goal: To judge the risk of measurement bias related to how PF was measured (differential measurement of PF related to the level of outcome).**			
Definition of the PF	A clear definition or description of 'PF' is provided (e.g., including dose, level, duration of exposure, and clear specification of the method of measurement).			
Valid and Reliable Measurement of PF	Method of PF measurement is adequately valid and reliable to limit misclassification bias (e.g., may include relevant outside sources of information on measurement properties, also characteristics, such as blind measurement and limited reliance on recall).			
	Continuous variables are reported or appropriate cut-points (i.e., not data-dependent) are used.			
Method and Setting of PF Measurement	The method and setting of measurement of PF is the same for all study participants.			
Proportion of data on PF available for analysis	Adequate proportion of the study sample has complete data for PF variable.			

Method used for missing data	Appropriate methods of imputation are used for missing 'PF' data.			
**PF Measurement Summary**	***PF*** **is adequately measured in study participants to sufficiently limit potential bias.**			
**4. Outcome Measurement**	**Goal: To judge the risk of bias related to the measurement of outcome (differential measurement of outcome related to the baseline level of PF).**			
Definition of the Outcome	A clear definition of outcome is provided, including duration of follow-up and level and extent of the outcome construct.			
Valid and Reliable Measurement of Outcome	The method of outcome measurement used is adequately valid and reliable to limit misclassification bias (e.g., may include relevant outside sources of information on measurement properties, also characteristics, such as blind measurement and confirmation of outcome with valid and reliable test).			
Method and Setting of Outcome Measurement	The method and setting of outcome measurement is the same for all study participants.			
**Outcome Measurement Summary**	***Outcome of interest*** **is adequately measured in study participants to sufficiently limit potential bias.**			
**5. Study Confounding**	**Goal: To judge the risk of bias due to confounding (i.e. the effect of PF is distorted by another factor that is related to PF and outcome).**			
Important Confounders Measured	All important confounders, including treatments (key variables in conceptual model: LIST), are measured.			
Definition of the confounding factor	Clear definitions of the important confounders measured are provided (e.g., including dose, level, and duration of exposures).			
Valid and Reliable Measurement of Confounders	Measurement of all important confounders is adequately valid and reliable (e.g., may include relevant outside sources of information on measurement properties, also characteristics, such as blind measurement and limited reliance on recall).			
Method and Setting of Confounding Measurement	The method and setting of confounding measurement are the same for all study participants.			
Method used for missing data	Appropriate methods are used if imputation is used for missing confounder data.			
Appropriate Accounting for Confounding	Important potential confounders are accounted for in the study design (e.g., matching for key variables, stratification, or initial assembly of comparable groups).			
	Important potential confounders are accounted for in the analysis (i.e., appropriate adjustment).			

**Study Confounding Summary**	**Important potential confounders are appropriately accounted for, limiting potential bias with respect to the relationship between *PF* and *outcome*.**			
**6. Statistical Analysis and Reporting**	**Goal: To judge the risk of bias related to the statistical analysis and presentation of results.**			
Presentation of analytical strategy	There is sufficient presentation of data to assess the adequacy of the analysis.			
Model development strategy	The strategy for model building (i.e., inclusion of variables in the statistical model) is appropriate and is based on a conceptual framework or model.			
	The selected statistical model is adequate for the design of the study.			
Reporting of results	There is no selective reporting of results.			
**Statistical Analysis and Presentation Summary**	**The statistical analysis is appropriate for the design of the study, limiting potential for presentation of invalid or spurious results.**			
